# Supplementary material for: Snake venom-defined fibrin architecture dictates fibroblast survival and differentiation
Source: Nat Commun. 2023 Feb 23;14:1029. doi: 10.1038/s41467-023-36437-9 (PMC9950370; doi:10.1038/s41467-023-36437-9)
Supplement: Supplementary file 1 — Supplementary Information [file 41467_2023_36437_MOESM1_ESM.pdf]

Supplementary Information for

Snake venom-defined fibrin architecture dictates fibroblast survival and differentiation

Zhao Wang[1], Jan Lauko[1], Amanda W. Kijas[1], Elliot P. Gilbert[1, 2], Petri Turunen[3], Ramanathan Yegappan[1], Dongxiu Zou[1], Jitendra Mata[2] and Alan E. Rowan[1]*

[1]Australian Institute for Bioengineering and Nanotechnology, The University of Queensland, St Lucia, QLD, 4072, Australia.
[2]Australian Centre for Neutron Scattering, Australian Nuclear Science and Technology Organisation, Lucas Heights, NSW, 2234, Australia.
[3]Microscopy Core Facility, Institute of Molecular Biology, Mainz, 55128, Germany.

Contents

Supplementary Figure 1. Gelation lag time of the fibrin networks formed by the snake venom-controlled and thrombin-initiated systems.

Supplementary Figure 2. SANS/USANS data of the snake venom-controlled and thrombin-initiated systems.

Supplementary Figure 3. Cleavage activity of the snake venom-controlled and thrombin-initiated systems.

Supplementary Figure 4. Influence of the addition of pFXIII and D004 on the mechanical properties and pore size of fibrin networks.

Supplementary Figure 5. FXIII variation in different fibrinogen samples.

Supplementary Figure 6. Relationship between the endogenous FXIII activities of different batches of fibrinogen and the gelation lag times of the formed fibrin networks.

Supplementary Figure 7. The incorporation of fibronectin into fibrin networks and its influence on fibrin network properties.

Supplementary Figure 8. Long-term stability of the fibrin networks controlled by textilinin.

Supplementary Figure 9. Permeability and diffusibility evaluation of the 3D fibrin networks.

Supplementary Figure 10. Biophysical details of fibrin networks used for cell experiments.

Supplementary Figure 11. Cell growth curve for fibroblasts in different fibrin networks.

Supplementary Figure 12. Morphological analysis of fibroblasts in different fibrin networks.

Supplementary Figure 13. The biocompatibility of recombinant ecarin and textilinin on human cell types of the skin.

Supplementary Figure 14. Transient fibroblast differentiation was detected by α-SMA and gene expression.

Supplementary Figure 15. Collagen expression of cultured fibroblasts/myofibroblasts in 3D fibrin networks.

Supplementary Figure 16. 2D control of fibroblast differentiation.

Supplementary Figure 17. Fibroblast F-actin expression and YAP nuclear translocation 3 days and 5 days after encapsulation.

Supplementary Figure 18. Focal adhesion formation of fibroblasts in different 3D fibrin networks.

Supplementary Figure 19. Summary of fibrinogen purification and quantification methods.

Supplementary Table 1. Batch information is listed with FXIII activity and FXIII concentration.

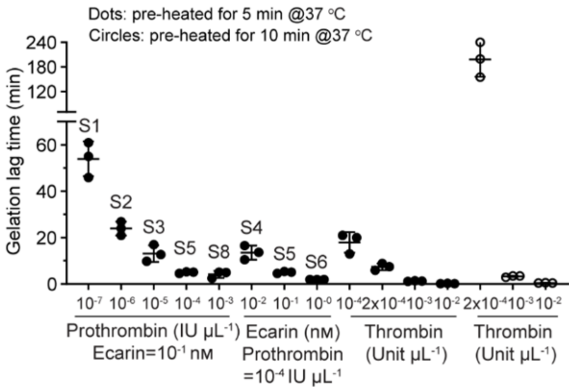

Supplementary Figure 1. Gelation lag time of the fibrin networks formed by the snake venom-controlled and thrombin-initiated systems. Gelation lag time was obtained from rheological measurements and defined as the time when the storage modulus G' of the network reached 5 Pa during polymerization. Ecarin/prothrombin was pre-heated at 37 °C for 5 min, while thrombin was pre-heated at 37 °C for either 5 min (dots) or 10 min (circles) before initiation. Data obtained from 3 independent measurements for each condition were included and shown as mean value ± SD (n=3).

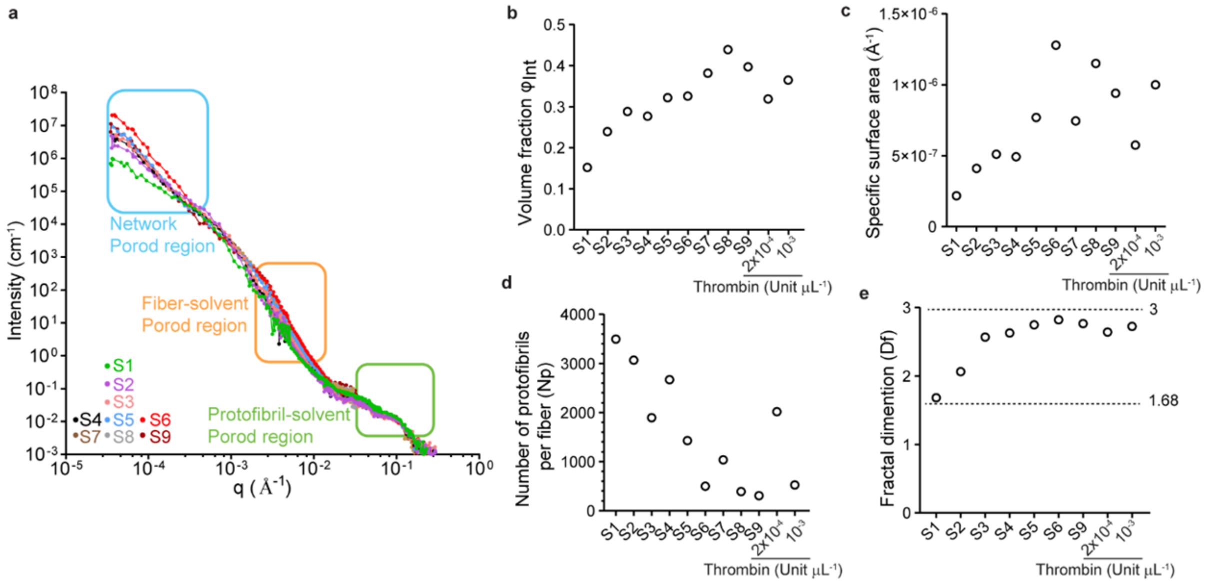

Supplementary Figure 2. SANS/USANS data of the snake venom-controlled and thrombin-initiated systems. a) Combined neutron scattering profiles of the snake venom-controlled fibrin networks (S1–S9) dissolved in 90% D_2O are shown. The scattering from three Porod regions (highlighted within the rectangles) was fitted and analyzed to yield details on the corresponding associated structures. b) Fiber density was determined from the volume fraction (φ_{Int}) of the protofibrils within fibers. c) Fiber surface area was determined by the specific surface area (S_v) by invariant analysis. d) The average number of protofibrils per fiber (Np) was analyzed using the diameter and volume fraction data. e) Fibrin branching was determined by fractal dimension (D_f) using fractal analysis. All fractal dimension values are between 1.68 and 3, indicated by dashed lines, while D_f =1.68 (5/3) is the signature value for fully unbranched swollen coils in the Porod region and D_f approaching 3 for branched networks. SANS/USANS data in (a–e) were obtained from 1 experiment for each condition (n=1).

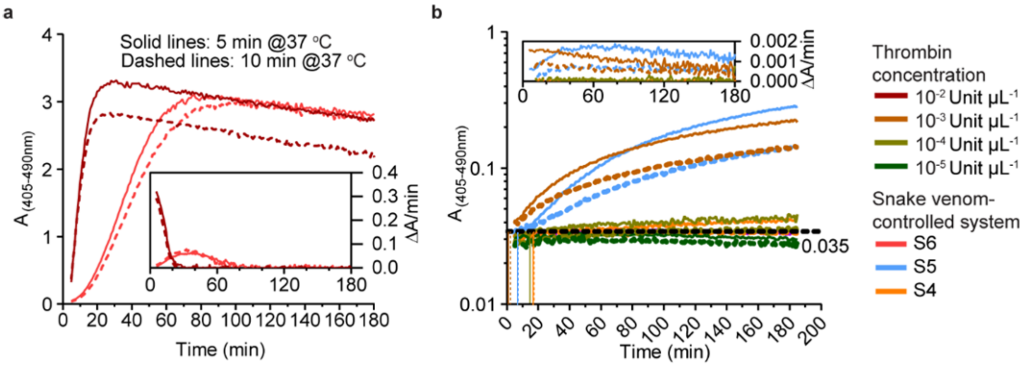

Supplementary Figure 3. Cleavage activity of the snake venom-controlled and thrombin-initiated systems. a, b) Cleavage absorbance reading $A_{(405\text{-}490nm)}$ and cleavage rate ΔA/min (insets) curves of thrombin (at 10^{-5} Unit μL^{-1}, 10^{-4} Unit μL^{-1}, 10^{-3} Unit μL^{-1}, and 10^{-2} Unit μL^{-1}) and ecarin/prothrombin (S4, S5, and S6) systems measured using a chromogenic substrate assay (S2238). Thrombin or ecarin/prothrombin was either pre-heated at 37 °C for 5 min (solid lines) or 10 min (dashed lines) before initiation. The time points when the absorbance reading reached the threshold of ~0.0035 were detected and labeled as vertical lines. Representative absorbance reading data in (a–b) were obtained from 1 experiment. The experiment was repeated 3 times with similar results.

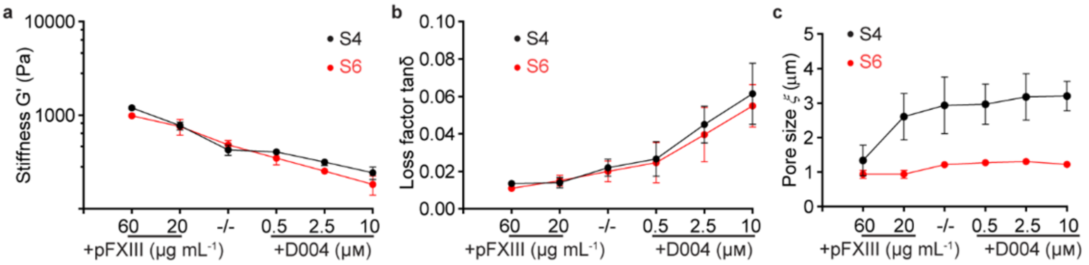

Supplementary Figure 4. Influence of the addition of pFXIII and D004 on the mechanical properties and pore size of fibrin networks. a–c) Tuning of mechanical properties, including matrix stiffness (a) and loss factor (b), and pore size of the networks S4 and S6 formed from the same batch (B4a in Supplementary Table 1) of fibrinogen by the addition of different concentrations of pFXIII and D004. The network without any addition (-/-) was set as control. Data obtained from 3 independent measurements for each condition are shown as mean value ± SD (n=3).

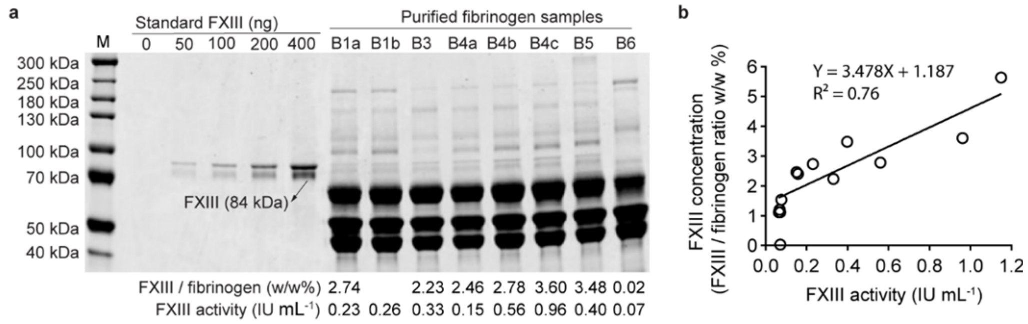

Supplementary Figure 5. FXIII variation in different fibrinogen samples. a) FXIII concentration in different batches of purified fibrinogen samples was quantified by reducing SDS-PAGE as the weight fraction in fibrinogen (w/w%). The standard FXIII protein (84 kDa, black arrow) served as a control group. Quantified values of FXIII concentrations are listed below each batch and compared with the FXIII activity measured by FXIII activity assays. FXIII activity was tested at 4 mg mL^{-1} of fibrinogen and presented as the activity per 1 mg mL^{-1} of fibrinogen. All data are shown as mean values. b) Relationship between FXIII concentration and FXIII activity in different batches of purified fibrinogen samples. A linear relationship was detected by linear regression analysis.

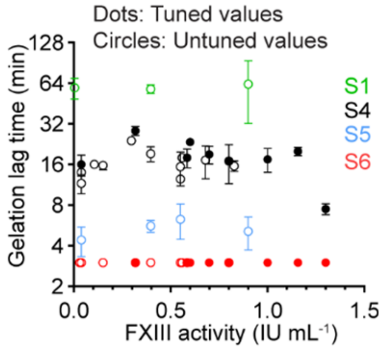

Supplementary Figure 6. Relationship between the endogenous FXIII activities of different batches of fibrinogen and the gelation lag times of the formed fibrin networks. Both the original untuned values (circles) and tuned values (dots) of gelation lag times of different fibrin networks were plotted as a function of endogenous FXIII activities of different batches of fibrinogen. Data obtained from 3 independent measurements for each condition are shown as mean values ± SD (n=3).

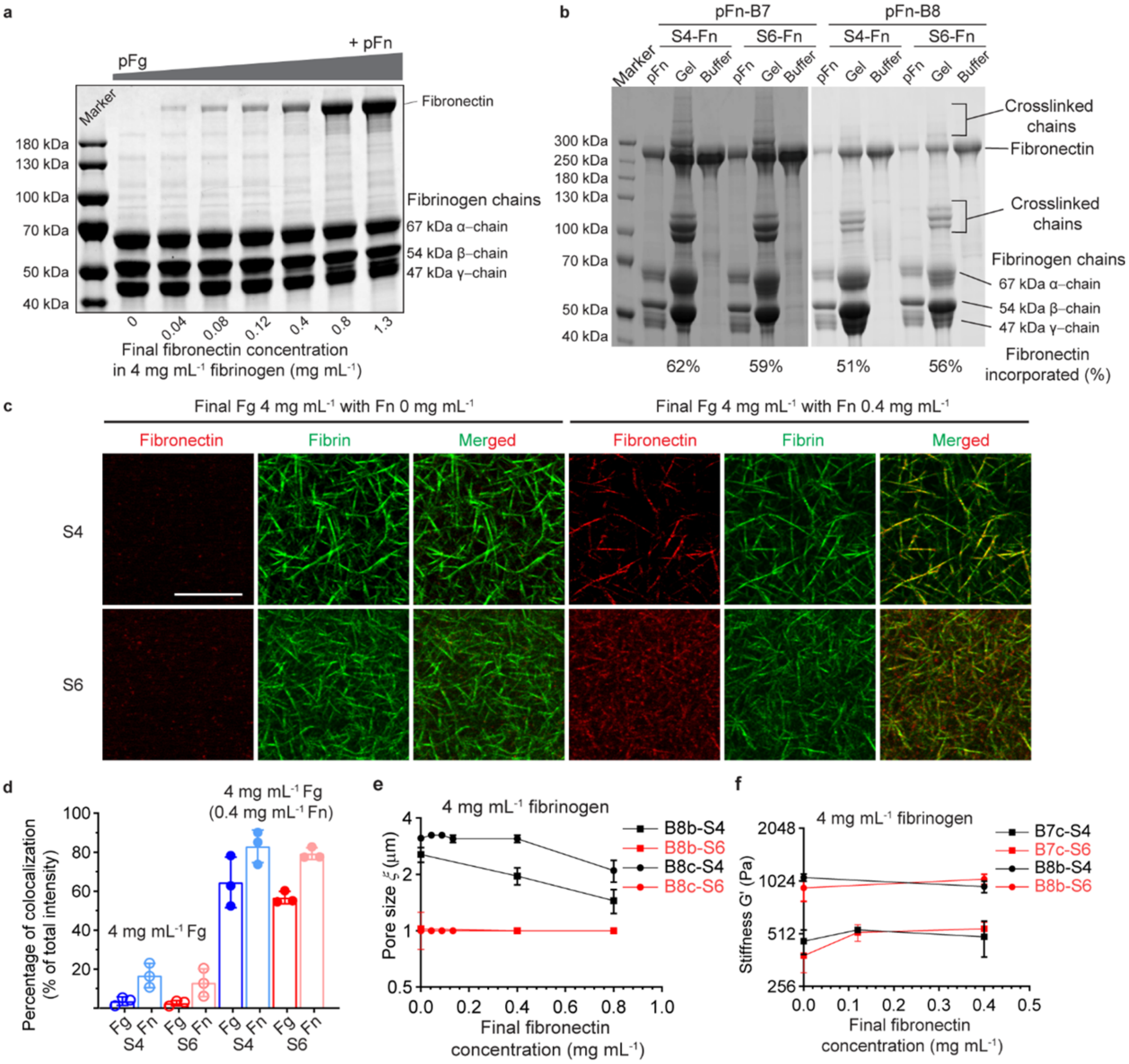

Supplementary Figure 7. The incorporation of fibronectin into fibrin networks and its influence on fibrin network properties. a) Final concentration of fibronectin in 4 mg mL^{-1} fibrinogen was tuned by the addition of purified fibronectin product (pFn) to purified fibrinogen (pFg). The final concentration of fibronectin (pFn-B8) was detected by Coomassie R250 staining of a reducing SDS-PAGE and semi-quantitative analysis by ImageJ. b) Incorporation of fibronectin (pFn-B7 and pFn-B8) into the fibrin networks evaluated by reducing SDS-PAGE. Samples before gelation (pFn), gel pellets after gelation (Gel), and the unincorporated fibronectin remaining in the buffer after gel removal (Buffer) were shown. The networks were formed under the same conditions as the S4 and S6 fibrin networks. The prominent protein bands are labeled in accordance with the estimated molecular weights, including uncharacterized crosslinked chains, fibronectin, and fibrinogen chains (α, β, and γ-chains). The percentage of incorporated fibronectin into the fibrin networks was determined relative to total fibronectin (pFn). Accordingly, around 50% of the fibronectin in the pFn product was incorporated, while the remaining fibronectin remained in the solution. c–d) Localization of fibronectin (red) and fibrin (green) in the fibrin networks (S4 and S6) with or without the addition of 0.4 mg mL^{-1} fibronectin by immunostaining. Confocal images are shown as single z-frame with an area of 40 μm × 40 μm. Scale bar, 20 μm. The colocalized signal intensities were analyzed and shown as the percentage of the total intensities. Fg, fibrinogen. Fn, fibronectin. e–f) Influence of the addition of purified fibronectin on the fibrin

pore size (e) and matrix stiffness (f). Two networks (S4 and S6) formed from different batches of purified fibrinogen were detected. Data shown in (d–f) were obtained from 3 biologically independent experiments and are shown as mean value ± SD (n=3).

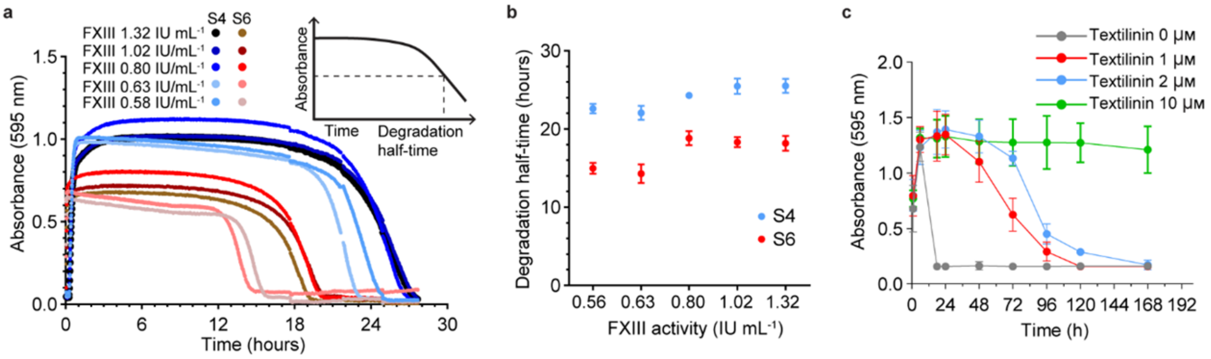

Supplementary Figure 8. Long-term stability of the fibrin networks controlled by textilinin. Characterization of degradation of fibrin networks (S4 and S6) with different FXIII activities (tuned by adding pFXIII or D004) measured by turbidimetry. Representative absorbance curves of the fibrin networks (S4 and S6) with different FXIII activities are shown in (a). Degradation half-time is defined as the time when the absorbance reading decreases to half of its maximum value. b) Degradation half-time of the formed S4 and S6 networks. c) Long-term stability of fibrin networks (S4 and S6) was determined by absorbance reading as a function of increasing concentrations of textilinin (0–10 μM). Data in (b–c) were obtained from 3 biologically independent experiments for each condition and are shown as mean value ± SD (n=3).

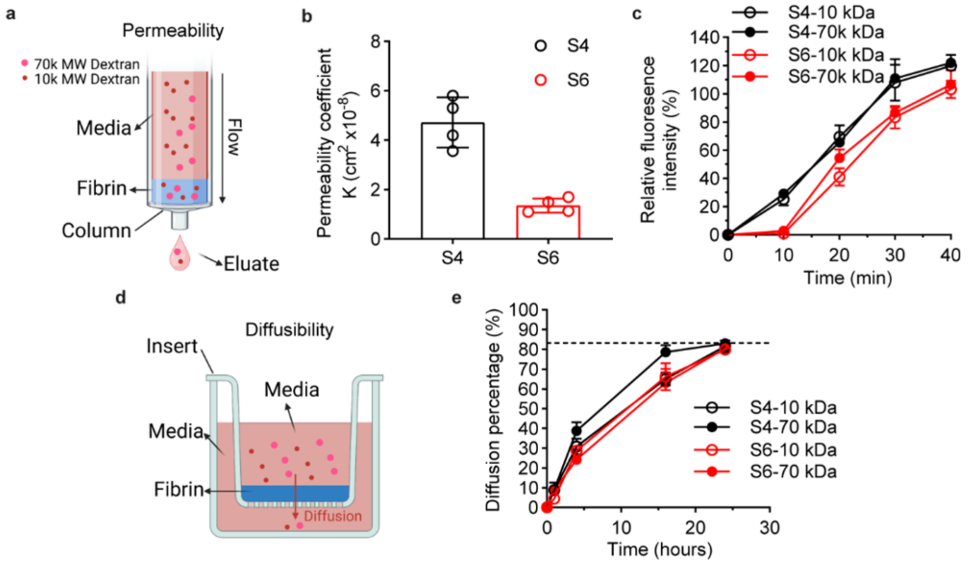

Supplementary Figure 9. Permeability and diffusibility evaluation of the 3D fibrin networks. a) Illustration of the 3D fibrin column permeability assay. The eluate volume was measured o ver time under hydrostatic pressure through the fibrin gel (150 µL), and the total volume of th e eluate was used to calculate the flow rate. b) Permeability coefficient K of S4 and S6 fibrin networks was calculated according to the flow rate of the permeability assay. Data were obtained from 4 independent experiments and shown as mean ± SD (n=4). c) The fluorescent i ntensities of 10 kDa and 70 kDa dextran in the eluate through the S4 or S6 fibrin networks we re measured and presented as relative intensities to the dextran in the starting media. The relati ve fluorescence intensities in the eluate were higher than the starting media, partly because of the concentrating effect (molecular sieving) as the dextran passed through the fibrin gels. d) Ill ustration of the fibrin diffusion assays evaluated. A cell culture insert with a 3 µm filter memb rane was used as the upper chamber, while a 24-well plate was the lower chamber. 50 µL of fi brin gel was evenly layered on the bottom of the insert. 2 hours after gelation at 37°C, 250 µL of DMEM serum-free media containing 10 µM textilinin with either 20 µg mL^{-1} 10 kDa dextran or 100 µg mL^{-1} 70 kDa dextran was added to the upper chamber. 1.25 mL of the same media without dextran was added to the lower chamber. Dextran diffused from the upper chamber to the lower chamber. e) Percentage of diffused dextran as a proportion of the total a mount of dextran. The dashed line indicates the percentage in the lower chamber after the completion of the diffusion. Data in (c) and (e) were obtained from 3 independent replicates and shown as mean value ± SD (n=3).

Panels a) and d) have been made in ©BioRender - biorender.com

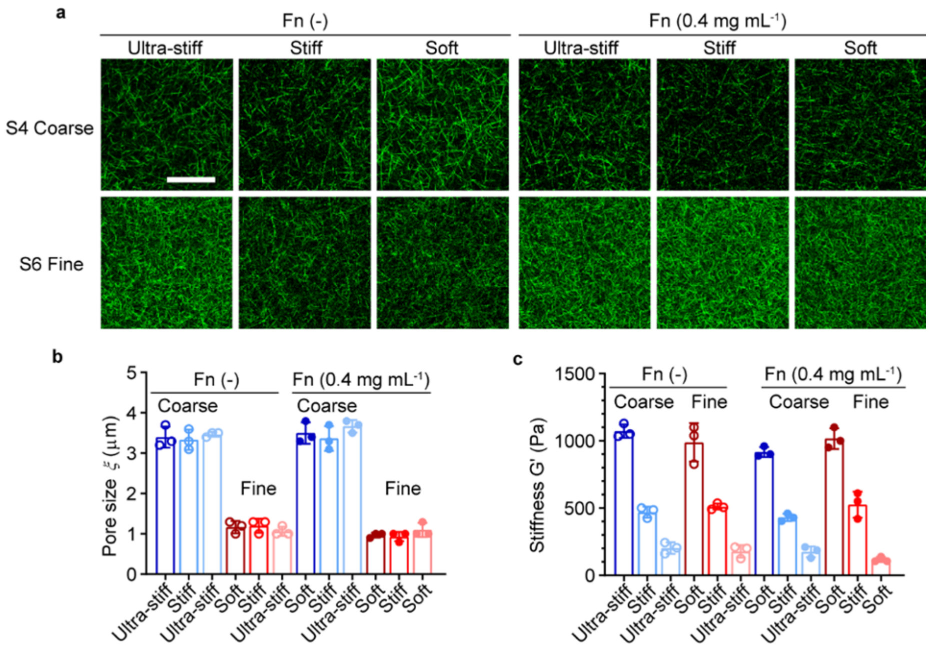

Supplementary Figure 10. Biophysical details of fibrin networks used for cell experiments. a) Confocal microscopy images of the fibrin networks (S4 and S6) with different stiffness tuned by adding D004 with or without 0.4 mg mL^{-1} of fibronectin. Images are shown as single z-frame with an area of 290 μm × 290 μm. Scale bar, 100 μm. b–c) Either pore size (b) or bulk stiffness (c) of these networks were measured from 3 independent replicates and shown as mean value ± SD (n=3).

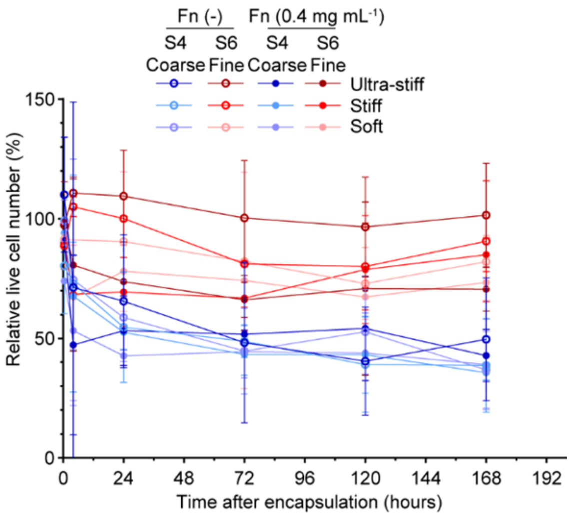

Supplementary Figure 11. Cell growth curve for fibroblasts in different fibrin networks. Live cell number (calcein+) was counted for the fibroblasts in different fibrin networks from 1 hour to 7 days after encapsulation. Data were obtained from 3 biologically independent experiments and are shown as mean value ± SD (n=3). Cell number for fibroblasts in stiff S6 network on day 1 served as control 100%.

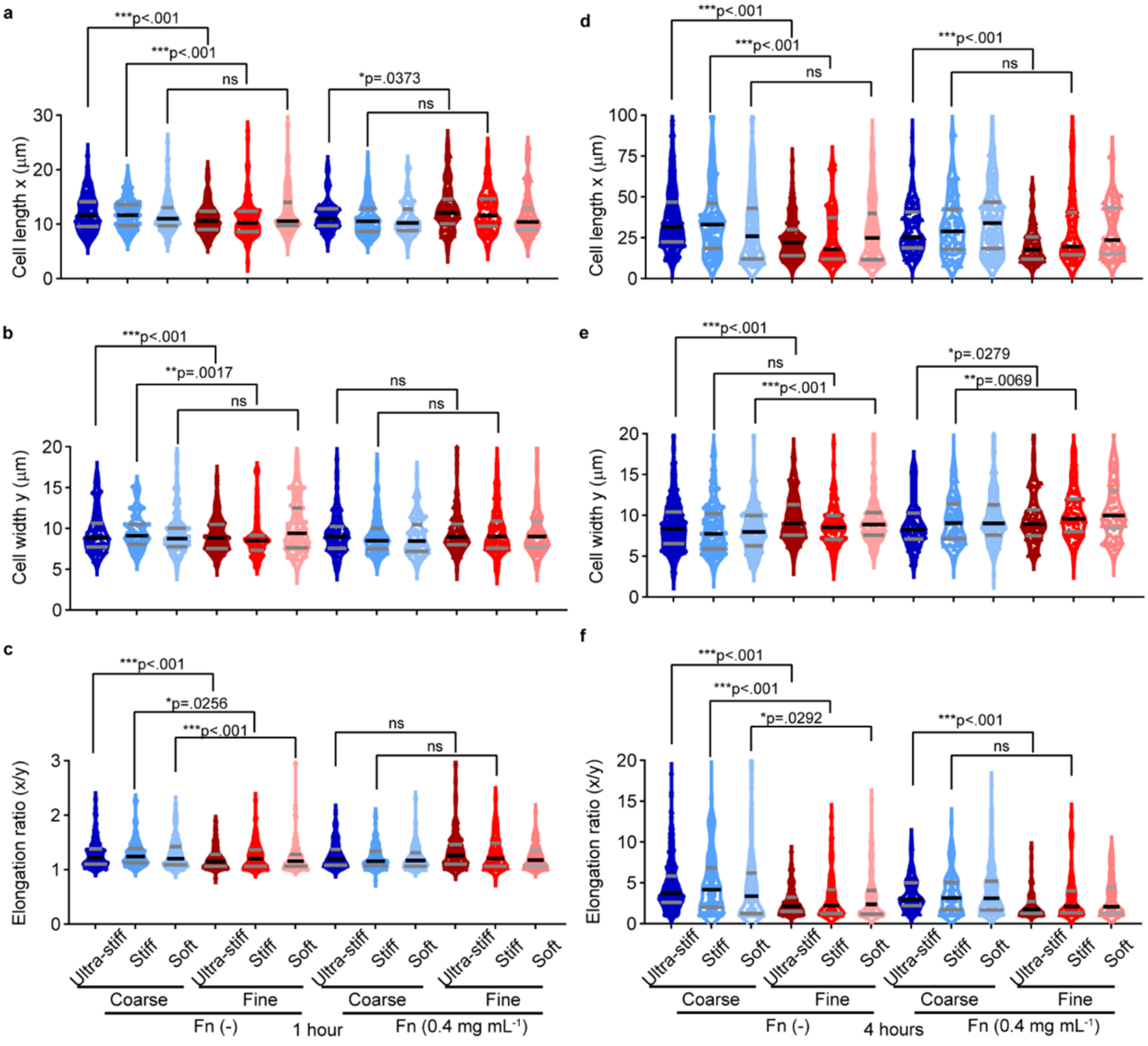

Supplementary Figure 12. Morphological analysis of fibroblasts in different fibrin networks. Cell length, cell width, and elongation ratio of individual fibroblasts grown in different fibrin networks were measured and plotted at early time points, including 1 hour (a–c) and 4 hours (d–f) after encapsulation. Data obtained from 3 biologically independent experiments were merged and shown as violin plots (n=3), two-tailed Wilcoxon signed-rank test. The black line indicates the median value, while the grey lines indicate quartiles. In (d–g), ns, not significant. *p < 0.05, **p < 0.01, ***p < 0.001.

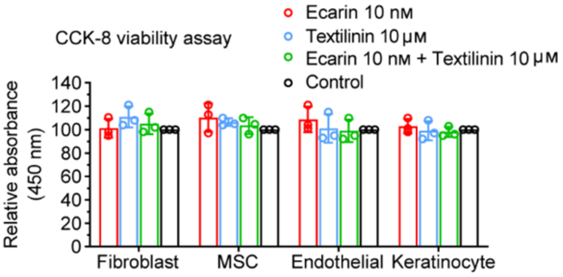

Supplementary Figure 13. The biocompatibility of recombinant ecarin and textilinin on human cell types of the skin. The cell viability was examined using the cell cytotoxicity kit-8 (CCK-8) after cells were treated with either 10 nM of ecarin, 10 μM of textilinin, or both for 7 days as compared to untreated controls. These cells investigated were primary fibroblasts, mesenchymal stem cells (MSC), the endothelial cell line EA.hy926, and HaCaT, a dermal keratinocyte cell line. Data from 3 biologically independent experiments are shown as mean value ± SD (n=3).

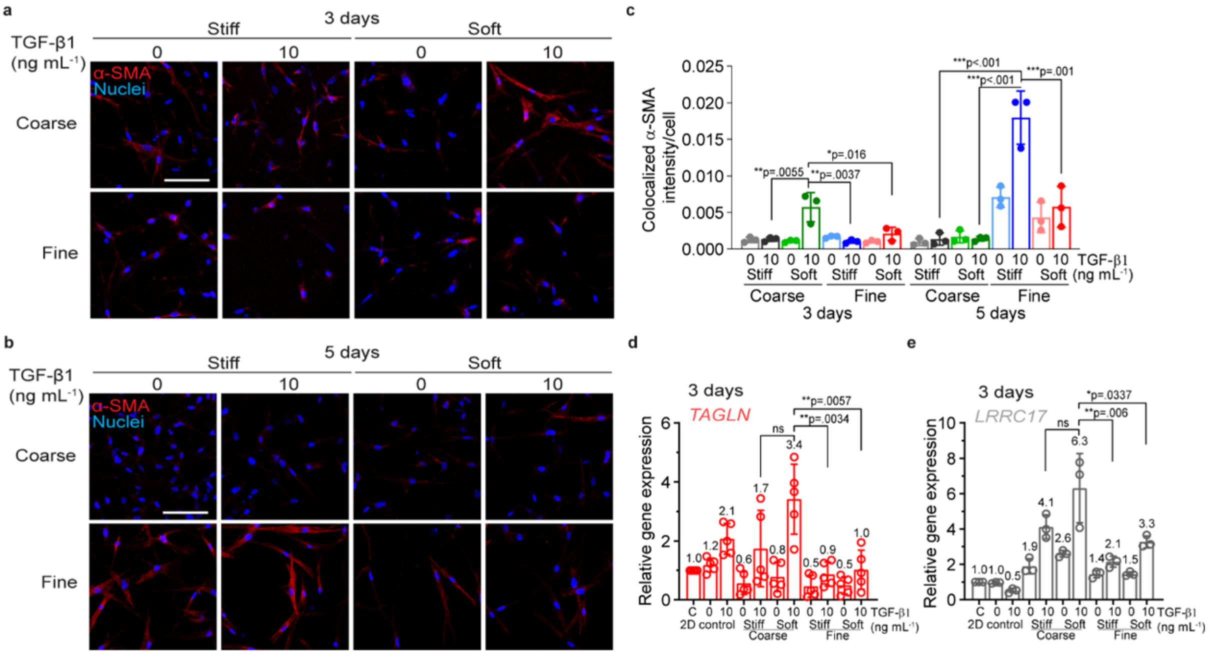

Supplementary Figure 14. Transient fibroblast differentiation was detected by α-SMA and gene expression. a, b) Confocal microscopy images of fibroblasts with α-SMA immunostaining (red) 3 days (a) or 5 days (b) after encapsulation. Images are shown as a maximum image projection (290 μm × 290 μm area with 20 μm thickness). Scale bar, 100 μm. c) Quantification of the colocalized α-SMA expression in different networks. Data from 3 biologically independent experiments were shown as mean value ± SD. Statistical analysis was performed by two-way ANOVA followed by Tukey's correction. n=3. ns, not significant. d–e) Myofibroblast-specific gene expression (*TAGLN* and *LRRC17*) analysis of fibroblast after 3 days of encapsulation. Dissociated fibroblast pellets on day 0 (denoted as C in the figure) or the fibroblasts cultured on 2D substrates served as control groups. Data were obtained from 5 (for *TAGLN*) or 3 (for *LRRC17*) biologically independent experiments and are shown as mean value ± SD. ns, not significant. *$p < 0.05$, **$p < 0.01$, ***$p < 0.001$.

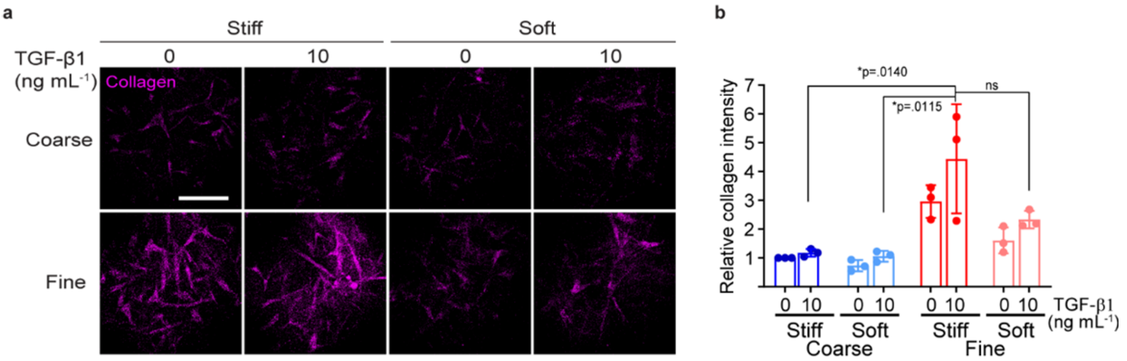

Supplementary Figure 15. Collagen expression of cultured fibroblasts/myofibroblasts in 3D fibrin networks. a) Collagen deposition was detected by collagen binding protein CNA35-mCherry in the different fibrin networks with and without TGF-β1 treatment after 7 days. Images are shown as a maximum image projection (290 μm × 290 μm area with 40 μm thickness). Scale bar, 100 μm. b) Total collagen production intensity was quantified and presented as relative collagen intensity to the stiff coarse untreated group (set to 1). Data were obtained from 3 biologically independent experiments and are shown as mean value ± SD. Statistical analysis was performed by two-way ANOVA (n=3) followed by Tukey's corrections. ns, not significant. *$p < 0.05$.

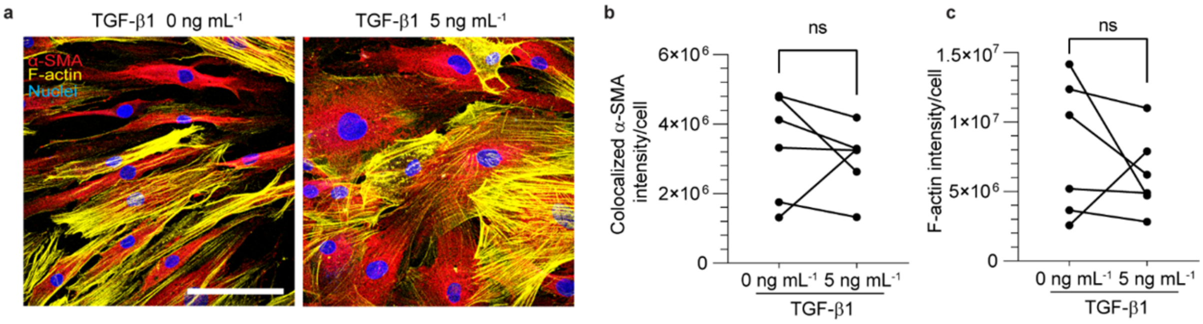

Supplementary Figure 16. 2D control of fibroblast differentiation. a) Confocal microscopy images of the fibroblasts cultured on 2D plates 7 days after treatment with TGF-β1. Both α-SMA (red) and F-actin (yellow) were stained. Images are shown as a maximum image projection (290 μm × 290 μm area with 20 μm thickness). Scale bar, 100 μm. b, c) Fluorescence intensity analysis of the α-SMA immunostaining (b) and the F-actin staining (c). Each matched dataset represents the data from one experiment. Data from 6 biologically independent experiments were analyzed using paired t-test (two-tailed, n=6). No significant difference in α-SMA or F-actin was detected between the TGF-β1-treated and untreated samples. ns, not significant.

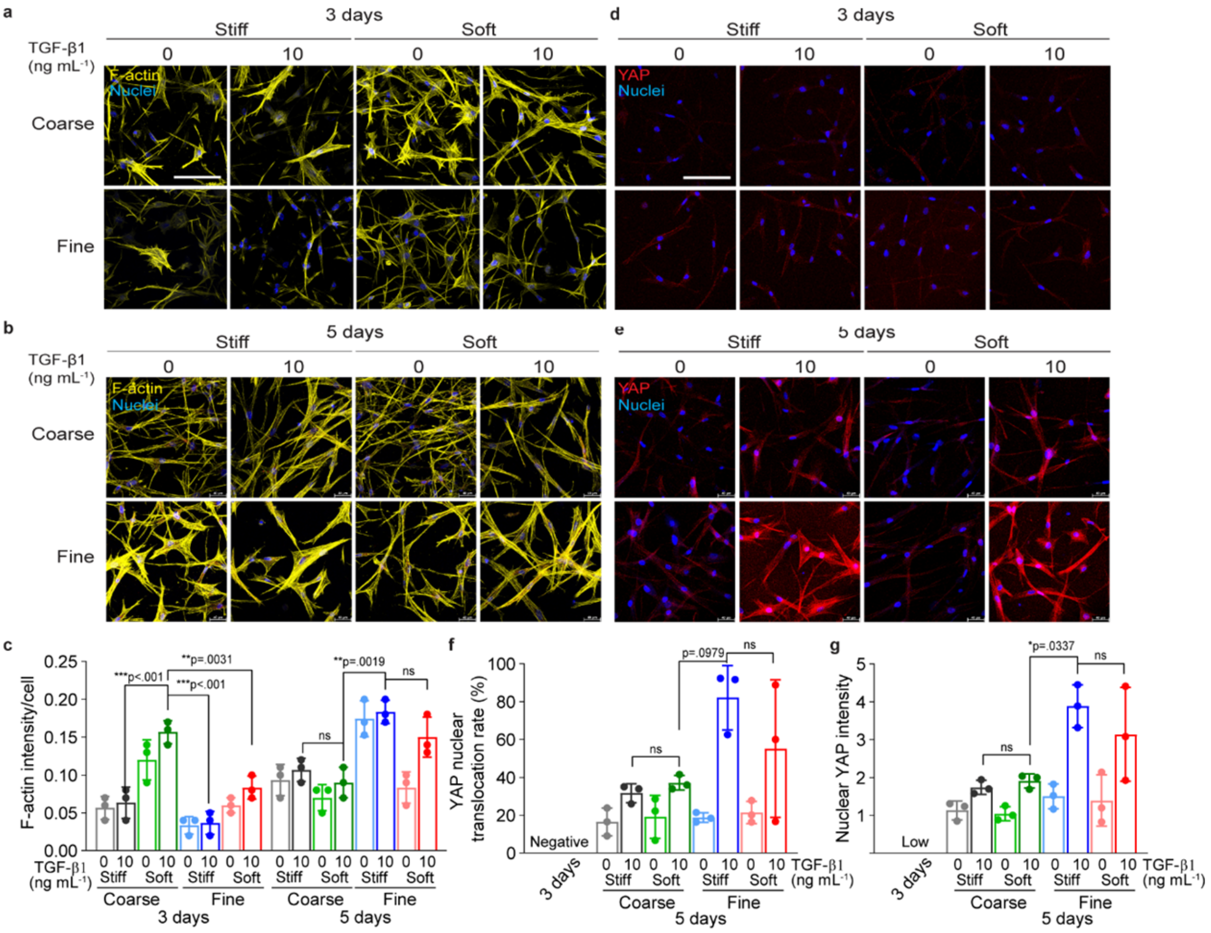

Supplementary Figure 17. Fibroblast F-actin expression and YAP nuclear translocation 3 days and 5 days after encapsulation. a–c) Confocal microscopic images of the fibroblasts with F-actin staining (yellow) 3 days (a) or 5 days (b) after encapsulation. Statistical analysis was performed by two-way ANOVA (c). $*p < 0.05$. d–g) Confocal microscopic images of the fibroblasts with YAP immunostaining (red) 3 days (d) or 5 days (e) after encapsulation. Quantification of YAP nuclear translocation rate (f) or Nuclear YAP intensity (g) was analyzed by two-way ANOVA followed by Tukey's correction. $**p < 0.01$. All the images are shown as a maximum image projection (290 μm × 290 μm area with 40 μm thickness). Scale bar, 100 μm. Data in (c, and f–g) were obtained from 3 biologically independent experiments and are shown as mean value ± SD (n=3).

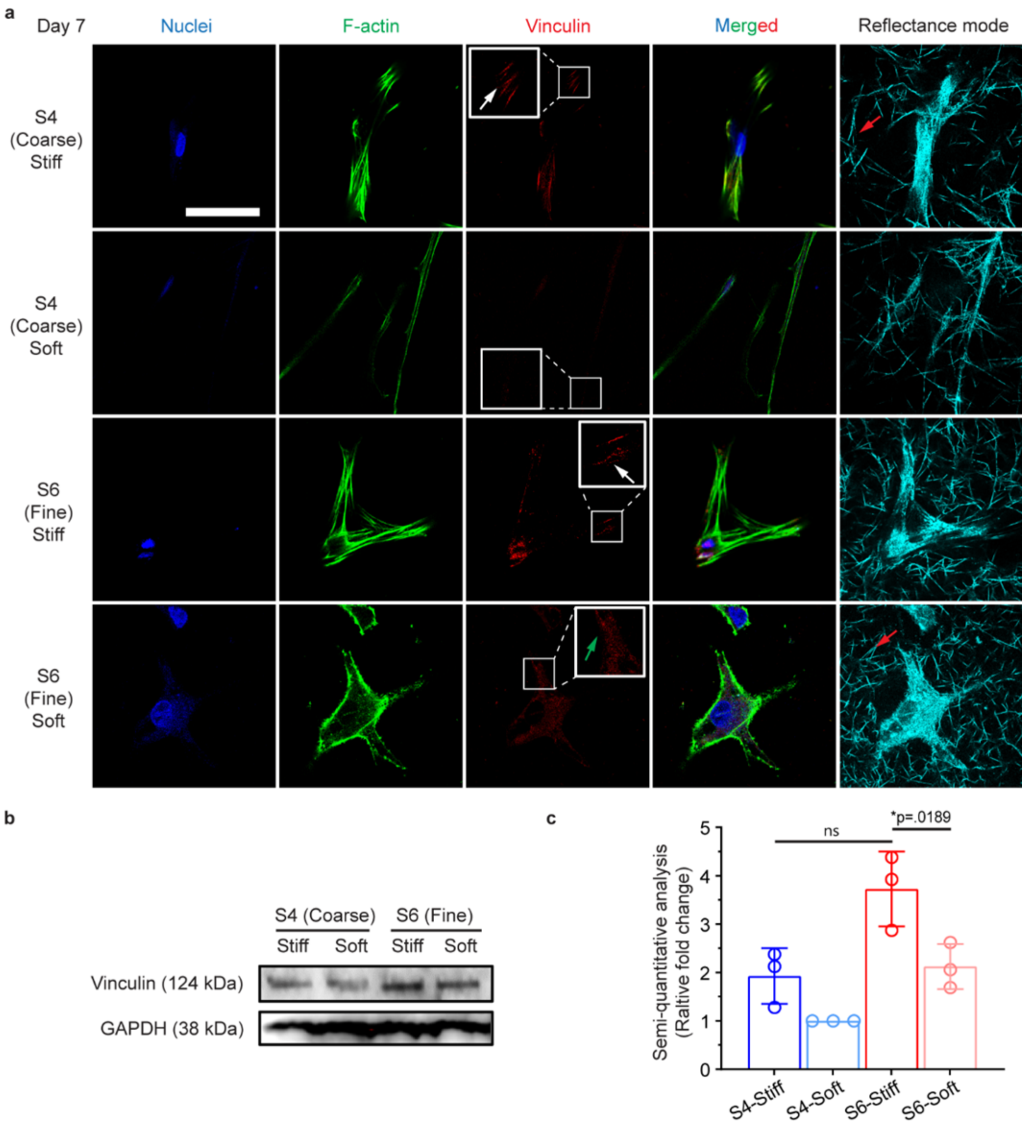

Supplementary Figure 18. Focal adhesion formation of fibroblasts in different 3D fibrin networks. a) Confocal microscopic images of focal adhesions of fibroblasts in different 3D fibrin networks were imaged by immunostaining. Cells were co-stained with F-actin and vinculin 7 days after treatment with TGF-β1. Images under reflectance mode were made in addition to visualize cells and fibrin networks simultaneously. All images are shown as a single frame (50 µm × 50 µm). Scale bar, 20 µm. Insets show magnified views of boxed areas with representative structures. White arrows indicate vinculin structures comprising focal adhesion complexes. The green arrow shows vinculin expression without assembling into complexes. The red arrows indicate fibrin fibers. b–c) Western blot analysis of focal adhesion-related protein vinculin and housekeeping protein GAPDH. Protein expression was semi-quantitatively analyzed and presented as relative protein expressions to the S4-Soft group. Data from 3 biologically independent experiments were shown as mean value ± SD and analyzed by one-way ANOVA followed by Tukey's correction (n=3). ns, not significant. $*p < 0.05$.

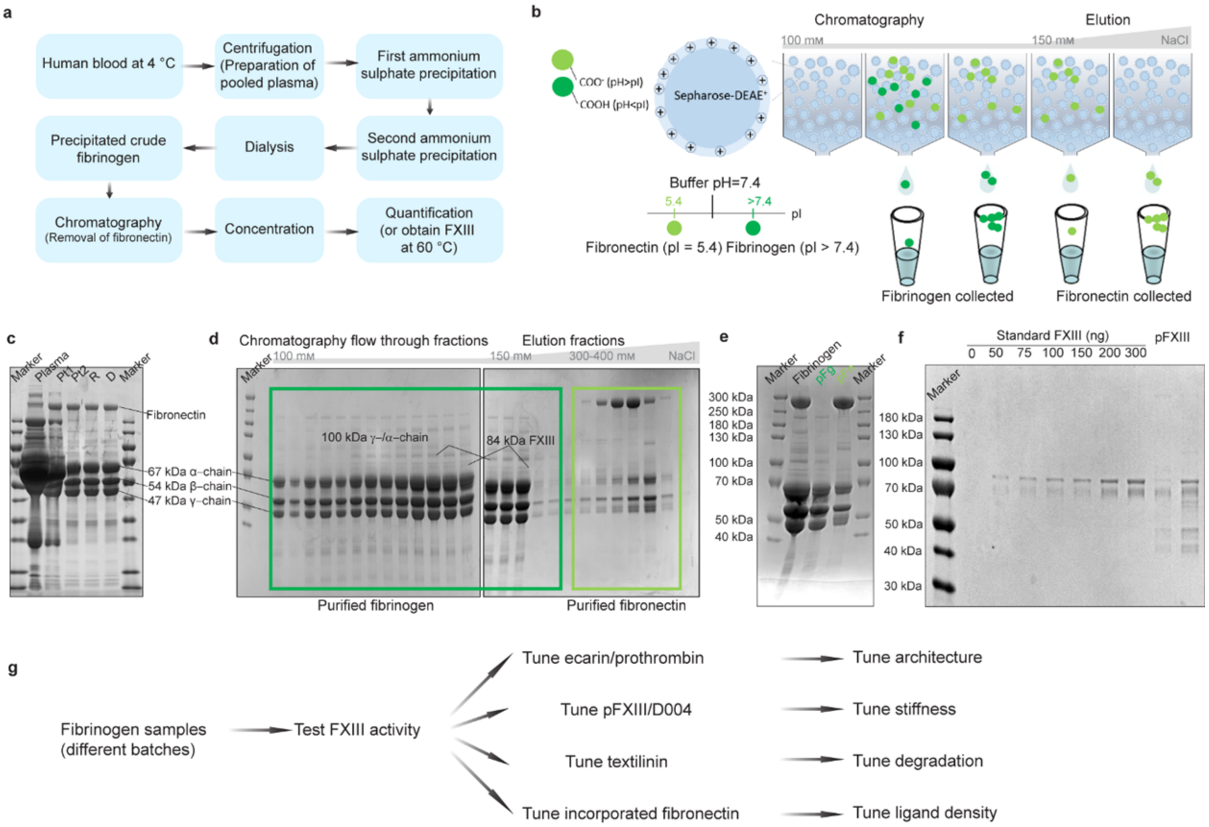

Supplementary Figure 19. Summary of fibrinogen purification and quantification methods. a) An overview of the fibrinogen, fibronectin, and FXIII purification protocol from the pooled human plasma. b) Schematic of the DEAE-Sepharose chromatography to collect fibrinogen and the removal of fibronectin. Under pH 7.4, fibrinogen (shown in dark green), with an isoelectric point (pI) higher than 7.4, has a positive charge and does not bind to the DEAE-Sepharose resin; it can be collected in the flow-through. The pI of fibronectin (shown in light green) is around 5.4. Fibronectin has a negative charge and binds to the resin at pH 7.4. By increasing the concentration of NaCl, the counter ions Cl^- deplete the charge on the fibronectin and result in the release of fibronectin (or fibrinogen-fibronectin complex). Therefore, fibronectin is collected in the elution buffer. c–e) Fibrinogen fractions quantification evaluated by reducing SDS-PAGE. Fibrinogen fractions during precipitation (c), elution (d), and after concentrating the fractions are shown in the dark green rectangle for purified fibrinogen (pFg) or in the light green rectangle for purified fibronectin (pFn) (e). Pt, fibrinogen pellet. f) Reducing SDS-PAGE images of the purified FXIII. g) Protocols to achieve reproducibility and tunability of biophysical properties of fibrin networks formed from different batches of fibrinogen samples. Reproducibility is achieved after characterizing the endogenous FXIII activity of each fibrinogen sample. Tunability of biophysical properties of fibrin, including architecture, stiffness, degradation, and ligand density, is achieved by tuning ecarin/prothrombin, pFXIII/D004, textilinin, and incorporated fibronectin individually.

**Batch No.**	**FXIII activity (IU mL^{-1})**	**FXIII concentration (FXIII/Fibrinogen ratio w/w%)**
B1a	0.23	2.74
B1b	0.26	Not detected
B2	Not detected	0.12
B3	0.33	2.23
B4a	0.15	2.46
B4b	0.56	2.78
B4c	0.96	3.60
B4d	0.83	Not detected
B5	0.40	3.48
B6	0.07	0.02
B7a	0.04	1.12
B7b	0.07	1.12
B7c	0.39	2.54
B7d	1.15	5.64
B8a	0.68	Not detected
B8b	0.80	Not detected
B8c	1.73	Not detected

Supplementary Table 1. Batch information is listed with FXIII activity and FXIII concentration.
