# Supplementary material for: Snake venom-defined fibrin architecture dictates fibroblast survival and differentiation
Source: Nat Commun. 2023 Feb 23;14:1029. doi: 10.1038/s41467-023-36437-9 (PMC9950370; doi:10.1038/s41467-023-36437-9)
Supplement: Supplementary file 3 — Reporting Summary [file 41467_2023_36437_MOESM3_ESM.pdf]

nature portfolio

Corresponding author(s): Alan E. Rowan

Last updated by author(s): Jan 22, 2023

Reporting Summary

Nature Portfolio wishes to improve the reproducibility of the work that we publish. This form provides structure for consistency and transparency in reporting. For further information on Nature Portfolio policies, see our Editorial Policies and the Editorial Policy Checklist.

Statistics

For all statistical analyses, confirm that the following items are present in the figure legend, table legend, main text, or Methods section.

n/a	Confirmed	
☐	☒	The exact sample size (*n*) for each experimental group/condition, given as a discrete number and unit of measurement
☐	☒	A statement on whether measurements were taken from distinct samples or whether the same sample was measured repeatedly
☐	☒	The statistical test(s) used AND whether they are one- or two-sided<br>*Only common tests should be described solely by name; describe more complex techniques in the Methods section.*
☒	☐	A description of all covariates tested
☐	☒	A description of any assumptions or corrections, such as tests of normality and adjustment for multiple comparisons
☐	☒	A full description of the statistical parameters including central tendency (e.g. means) or other basic estimates (e.g. regression coefficient) AND variation (e.g. standard deviation) or associated estimates of uncertainty (e.g. confidence intervals)
☐	☒	For null hypothesis testing, the test statistic (e.g. *F*, *t*, *r*) with confidence intervals, effect sizes, degrees of freedom and *P* value noted<br>*Give P values as exact values whenever suitable.*
☒	☐	For Bayesian analysis, information on the choice of priors and Markov chain Monte Carlo settings
☒	☐	For hierarchical and complex designs, identification of the appropriate level for tests and full reporting of outcomes
☒	☐	Estimates of effect sizes (e.g. Cohen's *d*, Pearson's *r*), indicating how they were calculated

Our web collection on statistics for biologists contains articles on many of the points above.

Software and code

Policy information about availability of computer code

Data collection	Confocal microscopic images were obtained using the Leica LAS X platform (V3.6.0). Rheology data were obtained using the Anton Paar RheoCompass V1.23. qPCR was performed using Bio-Rad CFX Real-Time PCR Detection System. SDS-PAGE images were obtained using ChemiDoc MP Imaging system (BioRad V6). Absorbance microplate reader was done using the i-control software (V1.1).
Data analysis	Confocal microscopic image analysis was done using the Leica LAS X platform (V3.6.0). Data plots and statistical analysis were performed using GraphPad Prism 9. Standard deviation was calculated using Microsoft Excel for Microsoft 365 (V16.0.13801.20240). Neutron scattering analysis was done using SASView Version 4. Protein scattering length density was calculated using the Biomolecular Scattering Length Density Calculator http://psldc.isis.rl.ac.uk/Psldc/. Immunofluoresence quantification was done using the BioImageXD 1.0 platform. SDS-PAGE image quantification was done using FIJI ImageJ (1.52p).

For manuscripts utilizing custom algorithms or software that are central to the research but not yet described in published literature, software must be made available to editors and reviewers. We strongly encourage code deposition in a community repository (e.g. GitHub). See the Nature Portfolio guidelines for submitting code & software for further information.

Data

Policy information about availability of data

All manuscripts must include a data availability statement. This statement should provide the following information, where applicable:

- Accession codes, unique identifiers, or web links for publicly available datasets
- A description of any restrictions on data availability
- For clinical datasets or third party data, please ensure that the statement adheres to our policy

The authors declare that all the data including the neutron scattering data used in this study are provided within the paper and in the Supplementary Information/ Source data file.

Human research participants

Policy information about studies involving human research participants and Sex and Gender in Research.

Reporting on sex and gender	The blood was provided by Australian Red Cross Lifeblood as de-identified, with no gender.
Population characteristics	The blood was provided by Australian Red Cross Lifeblood as de-identified, with no age or race.
Recruitment	The blood was provided by Australian Red Cross Lifeblood, Material Supply Deed 20-09QLD-05
Ethics oversight	The University of Queensland, Engineering, Architecture and Information Low and Negligible Risk Ethics Sub-Committee

Note that full information on the approval of the study protocol must also be provided in the manuscript.

Field-specific reporting

Please select the one below that is the best fit for your research. If you are not sure, read the appropriate sections before making your selection.

[x] Life sciences [ ] Behavioural & social sciences [ ] Ecological, evolutionary & environmental sciences

For a reference copy of the document with all sections, see nature.com/documents/nr-reporting-summary-flat.pdf

Life sciences study design

All studies must disclose on these points even when the disclosure is negative.

Sample size	Sample sizes were determined based on similar studies: DOI: 10.1073/pnas.1919394117; DOI: 10.1126/sciadv.aao4881.
Data exclusions	No data was excluded.
Replication	All the data in this study were the results from independent experiments (n≥3) with the number of replicates detailed in the specific figure legends. All attempt at replication for the snake venom-controlled fibrin system were successful and reproducible. For the traditional thrombin-initiated fibrin system, a severe variation was observed and not reproducible results including the network formation failure and a large variation in structural properties were seen and reported in the study.
Randomization	Fibrinogen (during material preparation) and cells (during 3D cell encapsulation) were allocated randomly as both fibrinogen and cell fractions were prepared into different equivalent aliquots before fibrin formation. During fibrin formation, randomization cannot be achieved as the gelation lag times in different groups must be known for a successful formation. For the imaging of the cell experiments, different regions of the cells in the slide chambers were randomly chosen and imaged blindedly without analyzing or knowing the results. For the imaging and structural quantification of the fibrin networks, different regions of the fibrin network formed in the slide chambers were randomly chosen for imaging and analysis.
Blinding	Critical experiments including fibrin formation and cell experiments were first performed by the principle investigator without blinding because the gelation lag time of different groups must be known for a successful fibrin formation and cell encapsulation. However, for repeating experiments, independent investigators undertook sample preparation, allocation, data collection and data analysis blindedly without knowing the experiment details or having an expectation of the results. For neutron scattering experiments, independent investigators participated in the sample preparation, loading, data collection and analysis independently and blindedly. All the other experiments in this study were done at least one independent investigator with blinded sample allocation without knowing the experiment details or expectation of the results.

Reporting for specific materials, systems and methods

We require information from authors about some types of materials, experimental systems and methods used in many studies. Here, indicate whether each material, system or method listed is relevant to your study. If you are not sure if a list item applies to your research, read the appropriate section before selecting a response.

Materials & experimental systems

n/a	Involved in the study
☐	☒ Antibodies
☐	☒ Eukaryotic cell lines
☒	☐ Palaeontology and archaeology
☒	☐ Animals and other organisms
☒	☐ Clinical data
☒	☐ Dual use research of concern

Methods

n/a	Involved in the study
☒	☐ ChIP-seq
☒	☐ Flow cytometry
☒	☐ MRI-based neuroimaging

Antibodies

Antibodies used

Mouse anti-YAP1 antibody (63.7, Catalog number: sc-101199) was from Santa Cruz Biotechnology, USA.
Mouse anti-Actin, α-Smooth Muscle antibody (Catalog number: A2547) was from Sigma-Aldrich.
Goat anti-Mouse IgG (H+L) Alexa Fluor Plus 594 secondary antibody (Catalog number: A32742) was from Sigma-Aldrich.
Rabbit anti-Fibronectin Alexa Fluor 647 conjugate (#72943) was from Cell Signaling Technology.
Mouse anti-Vinculin antibody (#V9131) was obtained from Sigma-Aldrich.
Rabbit anti-GAPDH antibody (#10494-1-AP) was from Proteintech.
Goat anti-Rabbit HRP-conjugated antibody (#AQ132P) and Rabbit anti-Mouse HRP-conjugated antibody (#AQ160P) were purchased from Merck.

Validation

Mouse anti-YAP1 antibody has been validated for detecting human YAP1 by immunofluorescence staining (https://www.scbt.com/p/yap-antibody-63-7).
Mouse anti-Actin, α-Smooth Muscle antibody has been validated for detecting human α-SMA by immunofluorescence staining (https://www.sigmaaldrich.com/AU/en/product/sigma/a2547).
Mouse anti-Vinculin antibody has been validated for detecting human vinculin by immunofluorescence staining (https://www.sigmaaldrich.com/AU/en/product/sigma/v9131).
Rabbit anti-GAPDH antibody antibody has been validated for detecting human GAPDH by Western blot (https://www.ptglab.com/products/GAPDH-Antibody-10494-1-AP).

Eukaryotic cell lines

Policy information about cell lines and Sex and Gender in Research

Cell line source(s)

Human dermal fibroblasts (Catalogue number: PCS-201-012) and human endothelial cell line EA.hy926 (#CRL-2922) were purchased from the American Type Culture Collection (ATCC). Keratinocytes HaCat were obtained from Translational Research Institute (TRI), Australia. Human mesenchymal stem cells were obtained from StemCore, Australia.

Authentication

Since the primary cells or cell lines used in this study were purchased or obtained from ATCC, TRI, or StemCore, no authentication was required.

Mycoplasma contamination

The cell lines were negative for mycoplasma contamination before and among the cell experiments.

Commonly misidentified lines
(See ICLAC register)

No commonly misidentified cell line was used in the study.
